# Supplementary material for: PRL-2 phosphatase is required for vascular morphogenesis and angiogenic signaling
Source: Commun Biol. 2020 Oct 23;3:603. doi: 10.1038/s42003-020-01343-z (PMC7584612; doi:10.1038/s42003-020-01343-z)
Supplement: Supplementary file 1 — Supplementary Information [file 42003_2020_1343_MOESM1_ESM.pdf]

Supplementary Fig. 1

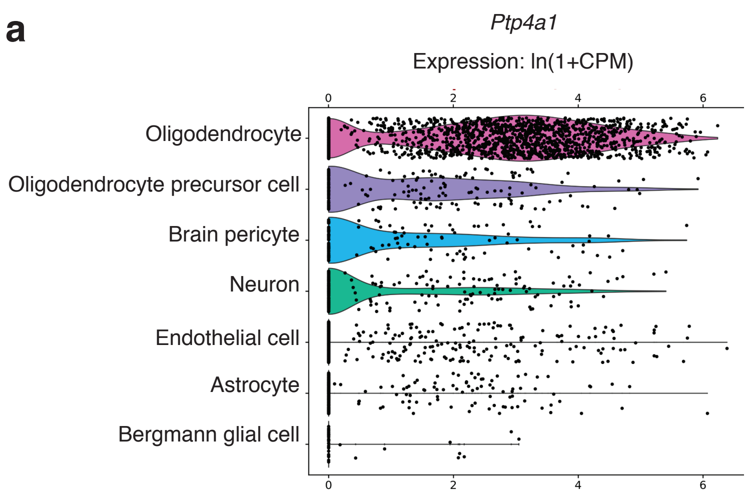

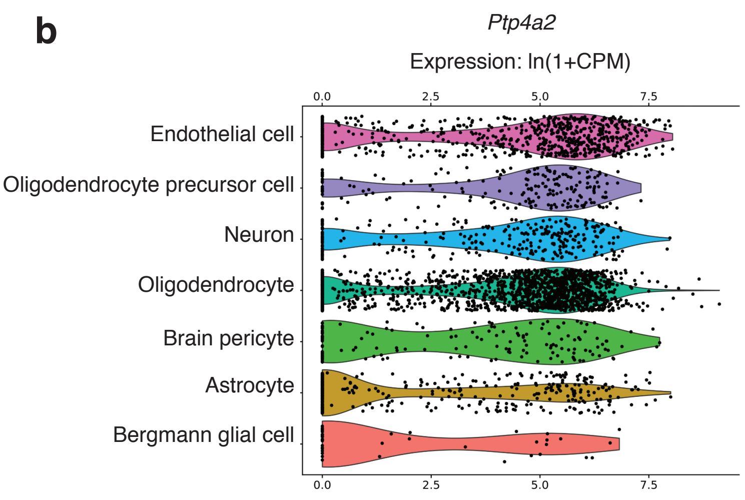

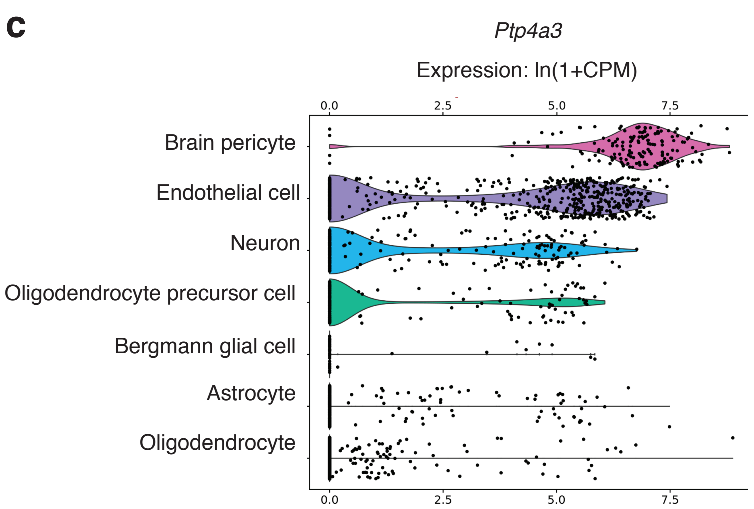

d

	Endothelial cell expression Ln(1+CPM)	
	Mean	Median
Ptp4a1	0.58	0.00
Ptp4a2	4.13	5.11
Ptp4a3	2.94	3.28

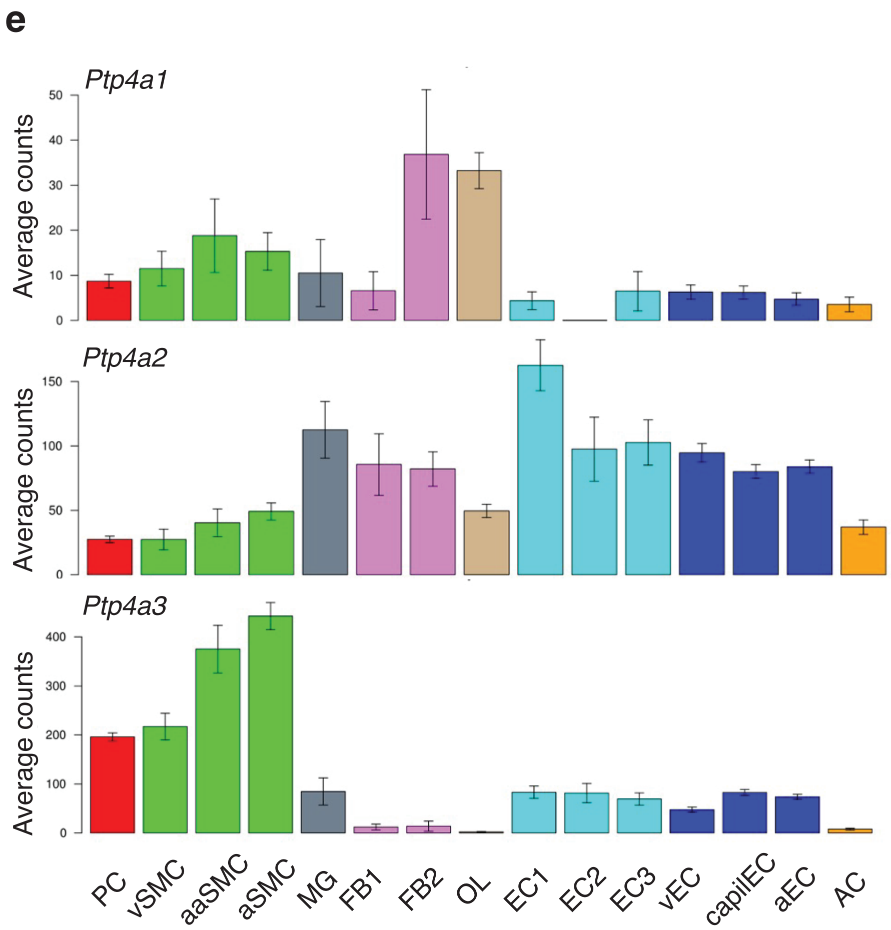

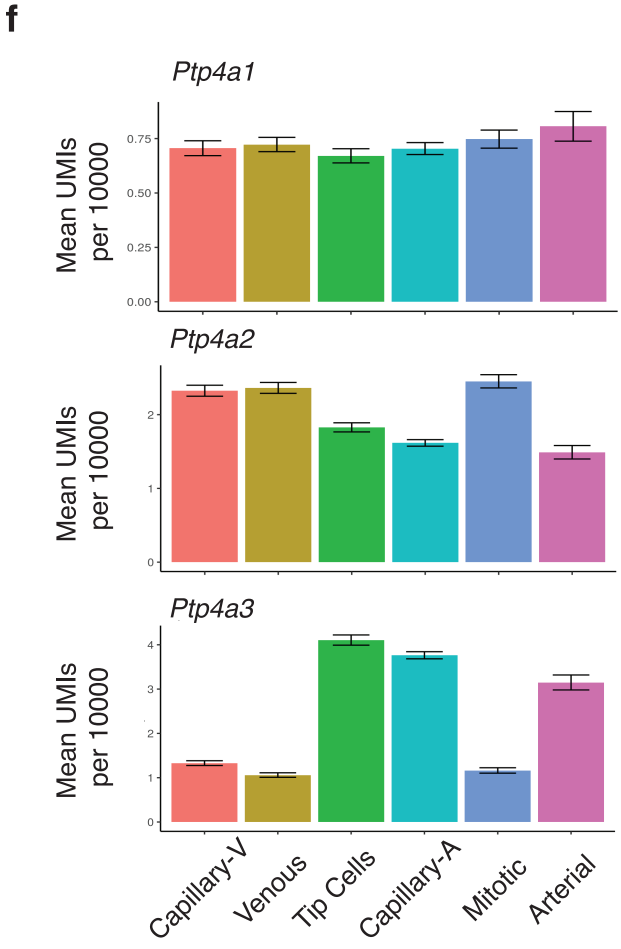

Supplementary Fig.1: (a) Analysis of Ptp4a1-3 expression single-cell transcriptomic data from the model organism Mus musculus (searchable database Tabula Muris https://tabula-muris.ds.czbiohub.org/)[52]. PRLs expression for each cell type sorted by FACs in brain (non-myeloid cells) can be visualized with violin plots. (b)Analysis of Ptp4a1-3 expression in the single cell RNA sequencing dataset[53,54]. (searchable database: http://betsholtzlab.org/VascularSingleCells/database.html) Average expression in each cluster [Brain data] PC - Pericytes; SMC - Smooth muscle cells; MG - Microglia; FB - Vascular fibroblast-like cells; OL - Oligodendrocytes; EC - Endothelial cells; AC - Astrocytes; v - venous; capil - capillary; a - arterial; aa - arteriolar; 1,2,3- subtypes. (c) Analysis of Ptp4a1,2,3 expression in Vascular Endothelial Cell Trans-omics Resource Database (VECTRDB) (https://markfsabbagh.shinyapps.io/vectrdb/[55]). PRLs gene expression in the developing CNS at single cell resolution. Single-cell RNA-seq on 3,946 FACS-purified GFP-positive endothelial cells from a P7 *Tie2-GFP* mouse brain. This dataset allow assessement Ptp4a1-3 expression in arterial, venous, capillary, mitotic, and tip subtypes of brain endothelial cells.

Supplementary Fig. 2

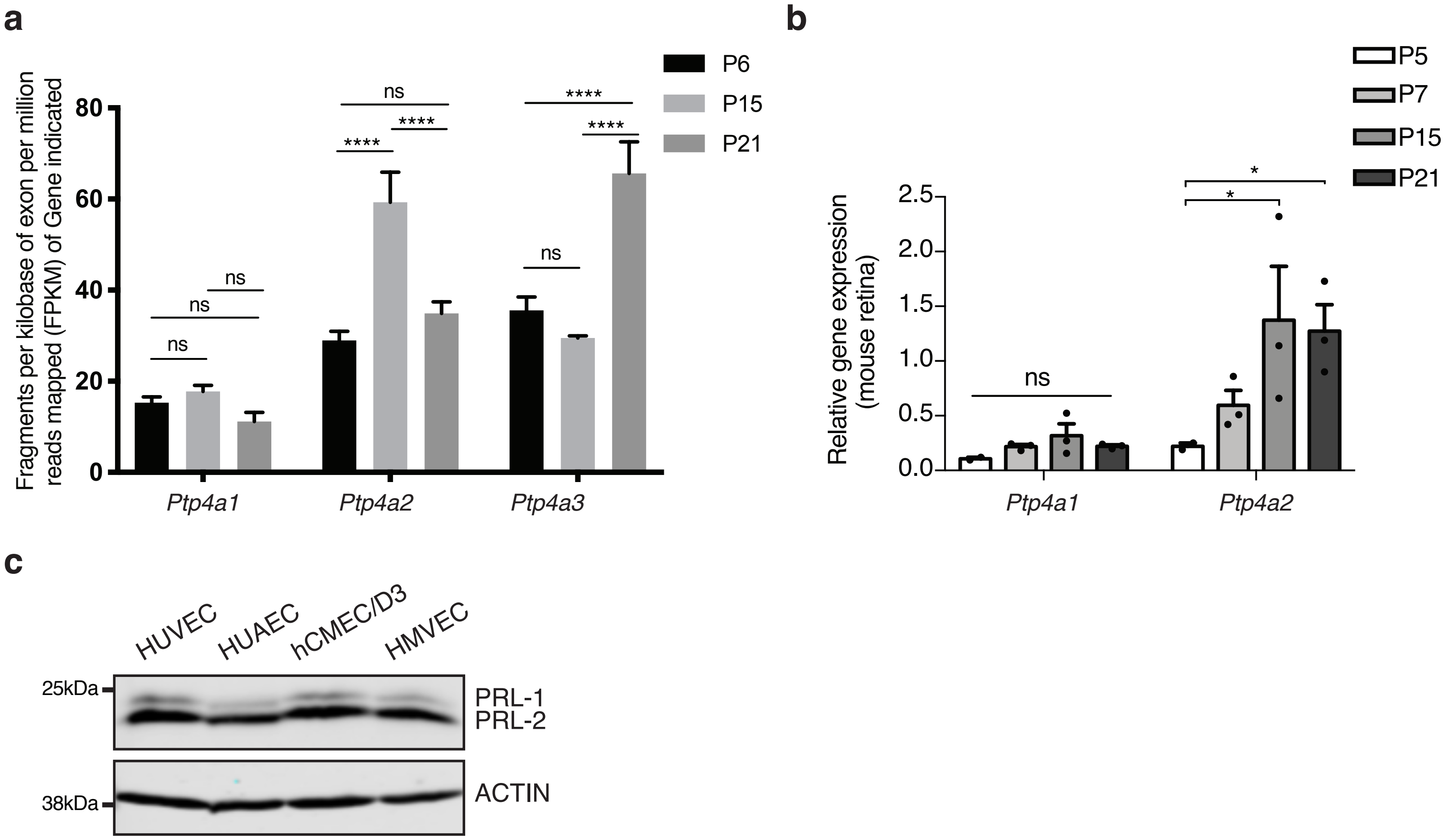

Supplementary Fig.2: (a) Analysis of online bulk RNA sequencing data from P6 to P21 mouse retinal endothelial cells[56] (Two-way ANOVA: *p-value<0.05, **p-value<0.01,***:p-value<0.001, ns: non-significant p-value>0.05). (b) qPCR analysis of mRNA isolated from whole mouse retina. Each individual data point represents a mouse (average of 2 retinas) (Two-way ANOVA: *p-value<0.05, ns: non-significant p-value>0.05). Error bars represent mean ± s.e.m. (c) Western-blot of several primary endothelial cell lines. Human Umbilical Vein Cell (HUVEC), Human Umbilical Artery Cell (HUAEC), Human Cerebral Microvascular Endothelial Cell (hCMEC/D3), Lung microvascular cells (HMVEC).

Supplementary Fig. 3

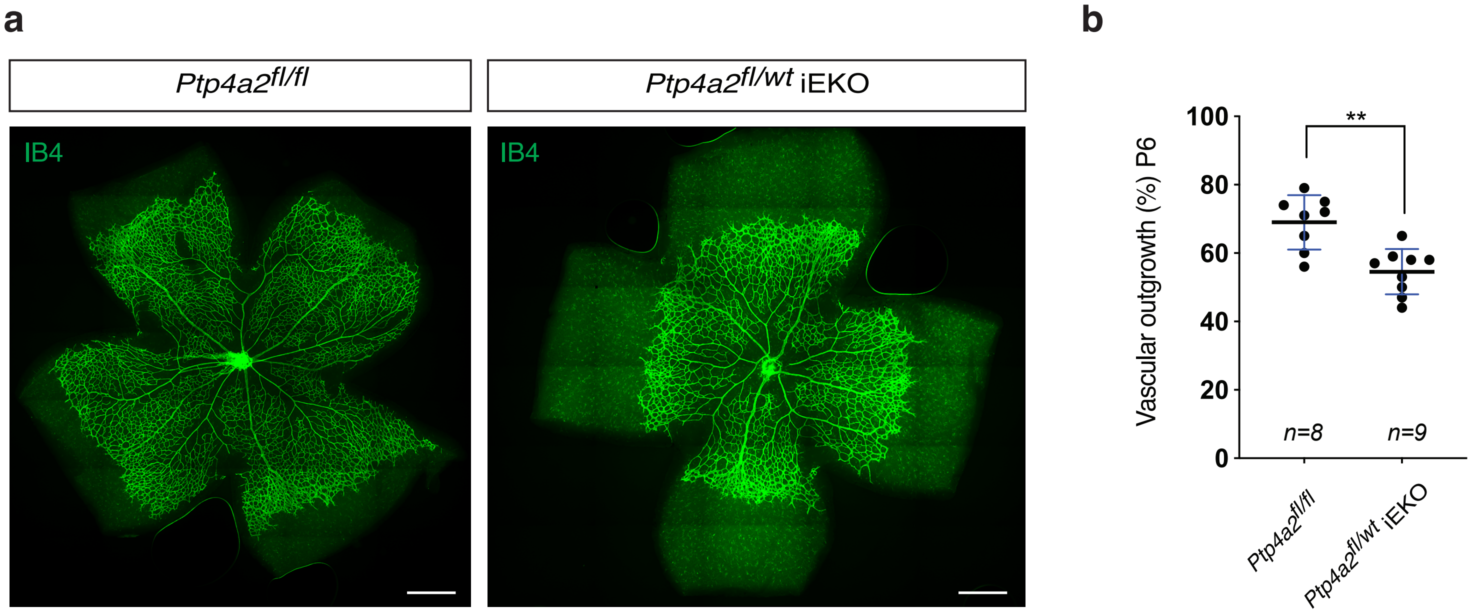

Supplementary Fig.3: (a) Retina whole-mount staining with isolectin B4 and (b) vascular outgrowth quantification in P6 $Ptp4a2^{fl/wt}$ and $Ptp4a2^{fl/wt}iEKO$. (Mann-Whitney U test: **: p-value<0.02). Each individual data point represents a mouse (average of 2 retinas). Error bars represent mean ± s.e.m. Scale bar: 500um

Supplementary Fig. 4

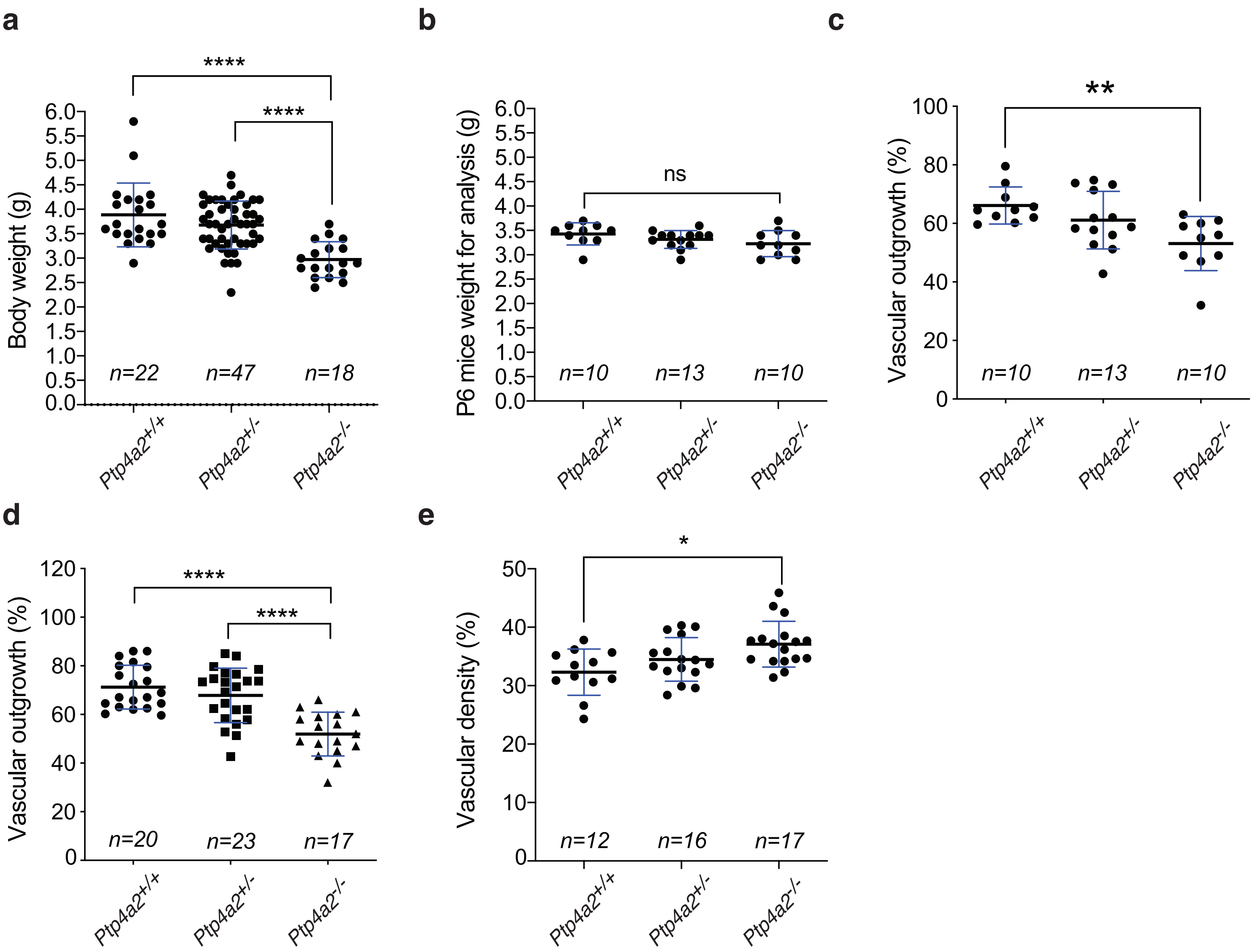

Supplementary Fig.4: (a) Body weight analysis of P6 *Ptp4a2*$^{+/+}$, *Ptp4a*$^{+/-}$ and *Ptp4a2*$^{-/-}$ mice. (One-way ANOVA: ****p-value<0.0001 ns: non-significant p-value>0.05) (b) Weight of mice selected for analysis on (c) (One-way ANOVA: ns: non-significant p-value>0.05). (c) Vascular outgrowth quantification of P6 *Ptp4a2*$^{+/+}$, *Ptp4a2*$^{+/-}$ and *Ptp4a2*$^{-/-}$ mice from similar weight (3.25g +/- 0.5g) (One-way ANOVA: **p-value<0.01, ns: non-significant p-value>0.05) (d,e) Vascular outgrowth and vascular density quantification of P6 *Ptp4a2*$^{+/+}$, *Ptp4a2*$^{+/-}$ and *Ptp4a2*$^{-/-}$ mice which show an intermediate phenotype for *Ptp4a2*$^{+/-}$ mice. All mice from all weight were included in this analysis. (One-way ANOVA: *: p-value<0.05 ****p-value<0.0001) Each individual data point represents a mouse. Error bars represent mean ± s.e.m.

Supplementary Fig. 5

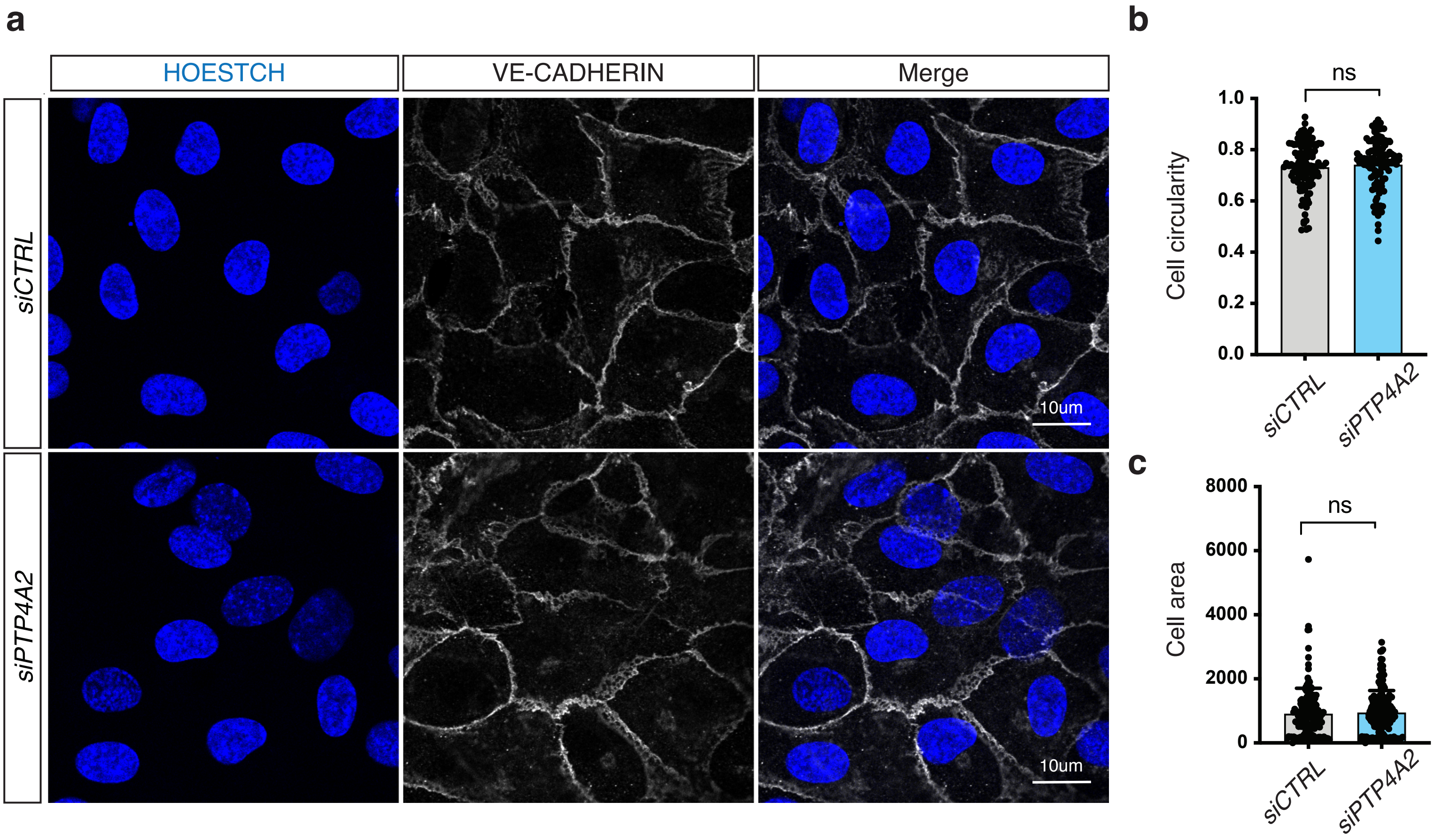

Supplementary Fig.5: (a) VE-CADHERIN staining in HUVECs after *siCTRL* or *siPTP4A2* treatment. Hoechst 33342 was applied to stain nuclei. Quantification of cell circularity (b) and cell area (c) of HUVEC after *siCTRL* or *siPTP4A2* treatment. At least 150 cells per group from n=3 independent experiment were analyzed (Mann-Whitney U test: ns: non-significant p-value>0.05).

Supplementary Fig. 6

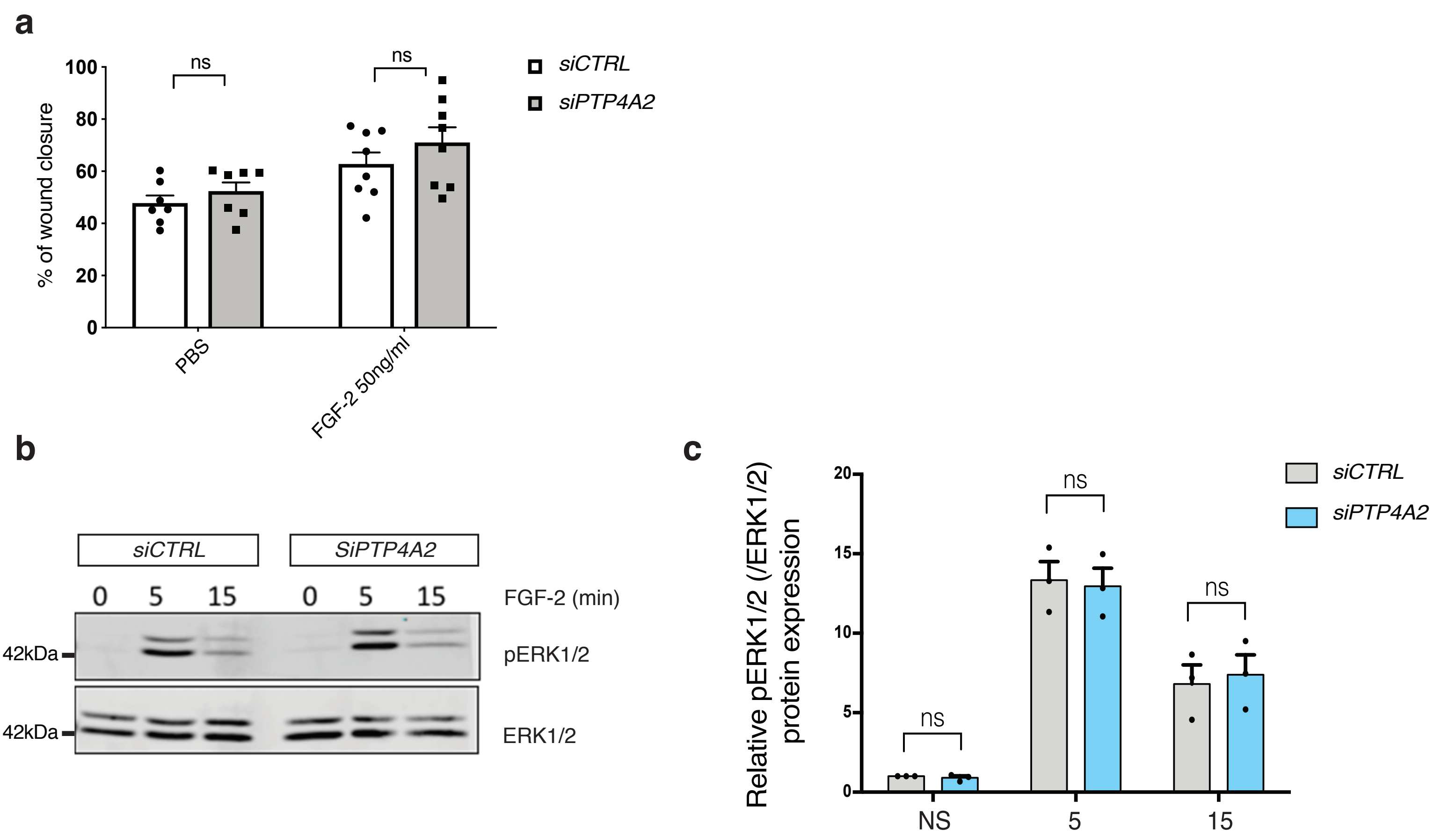

Supplementary Fig.6: (a) Scratch-wound assay performed on HUVEC monolayer 16h after scratch and with FGF-2 (50ng/ml). (Two-way ANOVA: ns: non-significant p-value>0.05). Each individual data point represents a biological replicat from n=3 independent experiments (b) Western blot and (c) quantification of HUVEC cells stimulated with FGF-2 after treatment with *siCTRL* or *siPTP4A2*. (Two-way ANOVA: ns: non-significant p-value>0.05). Error bars represent mean ± s.e.m.

Supplementary Fig. 7

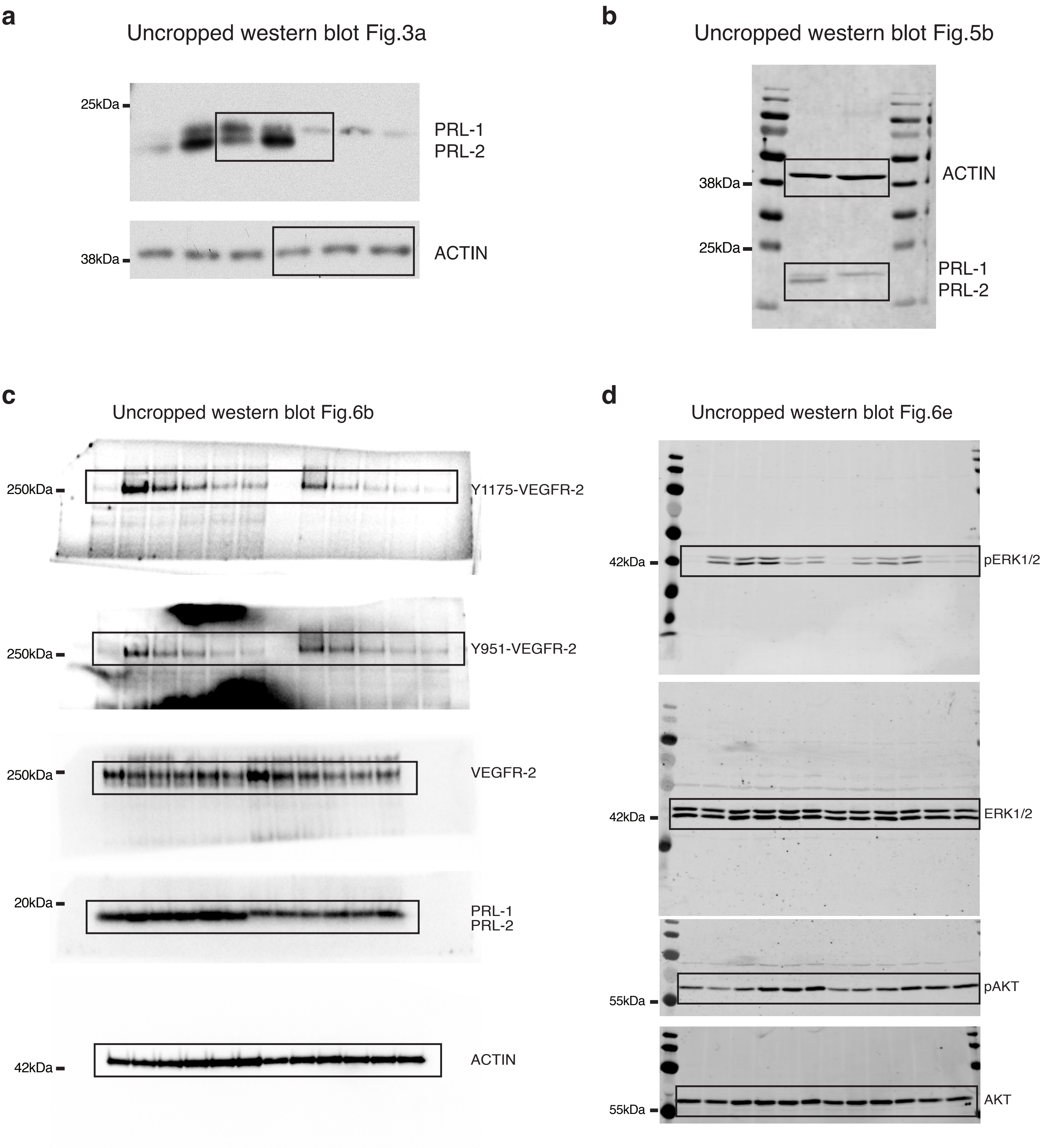

Supplementary Fig. 7: Uncropped western blot for (a) Fig.3a, (b) Fig.5b, (c) Fig6b, (d) Fig.6e

Supplementary Fig. 8

a

Uncropped western blot Fig.7b

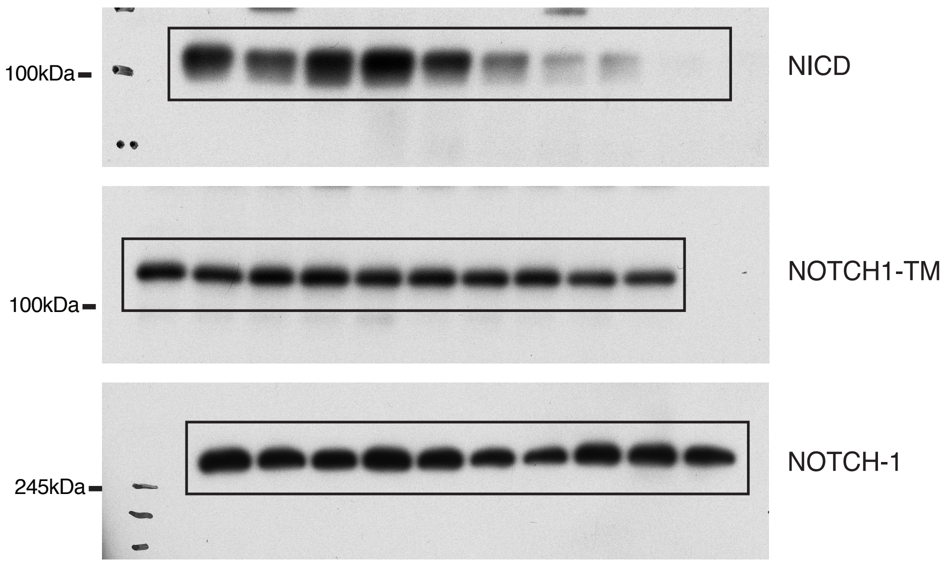

b

Uncropped western blot Supp Fig.2c

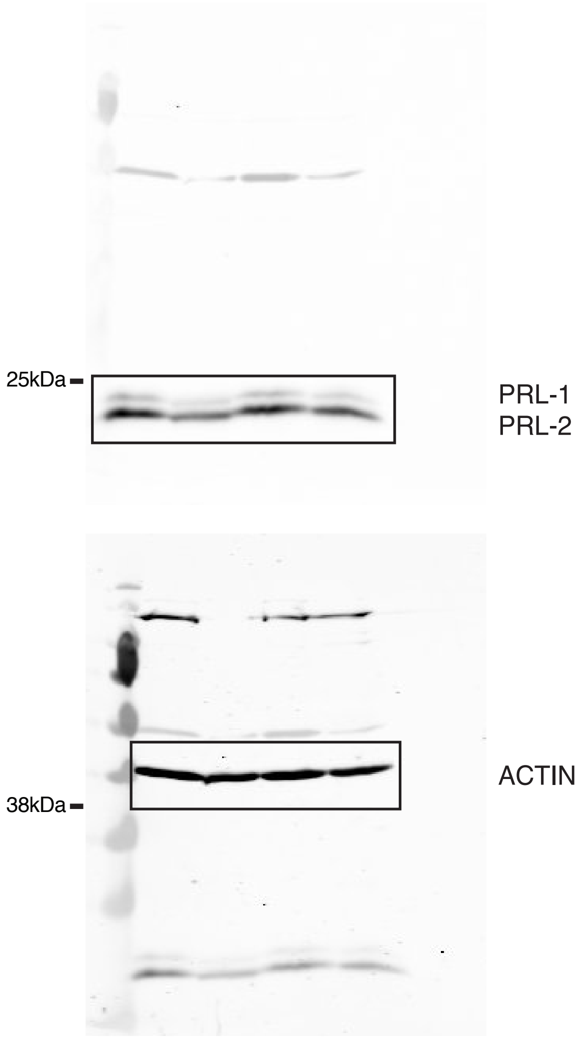

c

Uncropped western blot Supp Fig.6c

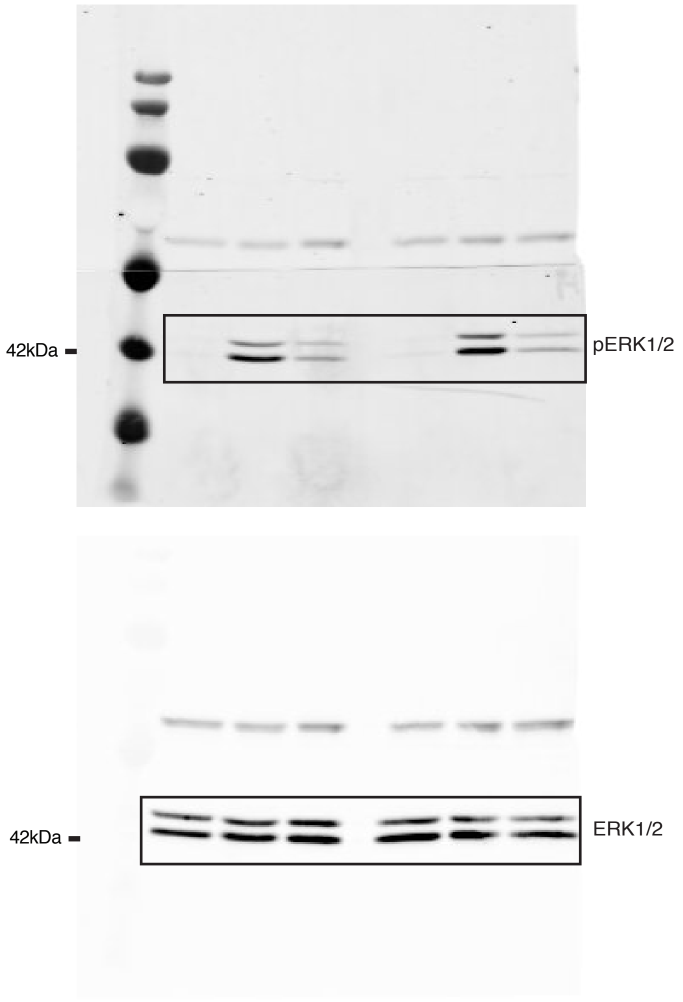

Supplementary Fig. 8: Uncropped western blot for (a) Fig.7b, (b) Supp Fig. 2c, (c) Supp Fig. 6c.
