# Supplementary material for: PRL-2 phosphatase is required for vascular morphogenesis and angiogenic signaling
Source: Commun Biol. 2020 Oct 23;3:603. doi: 10.1038/s42003-020-01343-z (PMC7584612; doi:10.1038/s42003-020-01343-z)
Supplement: Supplementary file 3 — Description of Additional Supplementary Files [file 42003_2020_1343_MOESM3_ESM.pdf]

Description of Additional Supplementary Files

File Name: Supplementary Data 1
Description: This file contains the qPCR primer sequence

File Name: Supplementary Data 2
Description: This file contains the raw data of the principal figures
