# Supplementary material for: PRL-2 phosphatase is required for vascular morphogenesis and angiogenic signaling
Source: Commun Biol. 2020 Oct 23;3:603. doi: 10.1038/s42003-020-01343-z (PMC7584612; doi:10.1038/s42003-020-01343-z)
Supplement: Supplementary file 6 — Reporting Summary [file 42003_2020_1343_MOESM6_ESM.pdf]

nature research

Corresponding author(s): Andreas BIKFALVI MD PhD

Last updated by author(s): Julv 22/2020

Reporting Summary

Nature Research wishes to improve the reproducibility of the work that we publish. This form provides structure for consistency and transparency in reporting. For further information on Nature Research policies, see Authors & Referees and the Editorial Policy Checklist.

Statistics

For all statistical analyses, confirm that the following items are present in the figure legend, table legend, main text, or Methods section.

n/a	Confirmed	
☐	☒	The exact sample size (n) for each experimental group/condition, given as a discrete number and unit of measurement
☐	☒	A statement on whether measurements were taken from distinct samples or whether the same sample was measured repeatedly
☐	☒	The statistical test(s) used AND whether they are one- or two-sided<br>*Only common tests should be described solely by name; describe more complex techniques in the Methods section.*
☐	☒	A description of all covariates tested
☐	☒	A description of any assumptions or corrections, such as tests of normality and adjustment for multiple comparisons
☐	☒	A full description of the statistical parameters including central tendency (e.g. means) or other basic estimates (e.g. regression coefficient) AND variation (e.g. standard deviation) or associated estimates of uncertainty (e.g. confidence intervals)
☒	☐	For null hypothesis testing, the test statistic (e.g. F, t, r) with confidence intervals, effect sizes, degrees of freedom and P value noted<br>*Give P values as exact values whenever suitable.*
☒	☐	For Bayesian analysis, information on the choice of priors and Markov chain Monte Carlo settings
☒	☐	For hierarchical and complex designs, identification of the appropriate level for tests and full reporting of outcomes
☒	☐	Estimates of effect sizes (e.g. Cohen's d, Pearson's r), indicating how they were calculated

Our web collection on statistics for biologists contains articles on many of the points above.

Software and code

Policy information about availability of computer code

Data collection	LightCycler 480 software, Leica SP8 software, ZEISS zen software
Data analysis	image-J, GraphPad Prism 5

For manuscripts utilizing custom algorithms or software that are central to the research but not yet described in published literature, software must be made available to editors/reviewers. We strongly encourage code deposition in a community repository (e.g. GitHub). See the Nature Research guidelines for submitting code & software for further information.

Data

Policy information about availability of data

All manuscripts must include a data availability statement. This statement should provide the following information, where applicable:

- Accession codes, unique identifiers, or web links for publicly available datasets
- A list of figures that have associated raw data
- A description of any restrictions on data availability

All data generated during this study are included in this article (main or supplementary information files). Additional informations can be obtained from the corresponding author upon reasonable request

Field-specific reporting

Please select the one below that is the best fit for your research. If you are not sure, read the appropriate sections before making your selection.

☒ Life sciences ☐ Behavioural & social sciences ☐ Ecological, evolutionary & environmental sciences

For a reference copy of the document with all sections, see nature.com/documents/nr-reporting-summary-flat.pdf

Life sciences study design

All studies must disclose on these points even when the disclosure is negative.

Sample size	We decided the sample size in order to reach a n number that we think is appropriate to show biological importance
Data exclusions	No data were excluded in this manuscripts except one out-layer point in figure 5g
Replication	In vivo experiment were done on at least n=4 mice In vitro experiment were done and repeated at least 3 times. For in vitro sprouting experiment a minimum of 24 beads were analysed
Randomization	Samples were not randomized
Blinding	several experiment analysis were validated blinded by a co-author

Reporting for specific materials, systems and methods

We require information from authors about some types of materials, experimental systems and methods used in many studies. Here, indicate whether each material, system or method listed is relevant to your study. If you are not sure if a list item applies to your research, read the appropriate section before selecting a response.

Materials & experimental systems

n/a	Involved in the study	
☐	☒	Antibodies
☐	☒	Eukaryotic cell lines
☒	☐	Palaeontology
☐	☒	Animals and other organisms
☒	☐	Human research participants
☒	☐	Clinical data

Methods

n/a	Involved in the study	
☒	☐	ChIP-seq
☒	☐	Flow cytometry
☒	☐	MRI-based neuroimaging

Antibodies

Antibodies used	p44/42 MAPK (p-ERK, cat# 9106, cell signaling), anti-p44/42 MAPK (total ERK, cat# 9102, cell signaling), anti-pAKT(Ser)473 (cat# 4060, cell signaling), anti-AKT (cat# 4691, cell signaling), Cleaved Notch1 (Val1744, cat# 4147, cell signaling), Notch 1 (D6F11, cat#4380, cell signaling), phospho-specific and total VEGFR-2 (total VEGFR-2, cat# #2479 cell signaling and Tyr1175 cat# #2478, Tyr1214 Tyr951 cat# #2471 cell signaling). Rabbit polyclonal anti–b-actin antibodies (cat# A2066, Sigma-Aldrich), Mouse monoclonal anti-PRL-2 (05-1583, EMD Millipore), Collagen IV (abcam), IB4 (Thermo Fisher), Ki67 (abcam), erg1/2/3 (abcam), asma (sigma).
Validation	Antibodies used in this studies were all reported and validated in the literature. We used them according to the datasheet providing from the supplier. We did not use any mouse antibodies to stain mouse tissue to avoid IgG unspecific staining

Eukaryotic cell lines

Policy information about cell lines

Cell line source(s)	All main experiment were done using HUVEC from Promocell. Cell line used in figure 1C were obtained and previously validated from Pr Bikfalvi's lab (HUAEC, hCMEC/D3, HMVEC)
Authentication	All cell lines used are already been used by several group
Mycoplasma contamination	HUVEC primary cell line were routinely check for mycoplama contamination and were never used after passage 5
Commonly misidentified lines (See ICLAC register)	n/a

Animals and other organisms

Policy information about studies involving animals; ARRIVE guidelines recommended for reporting animal research

Laboratory animals	PTP4A2-/- global knock out mice and PTP4A2 fl/fl-CDH5-CreERT2 endothelial specific mice were used in this study. Both male and female were used at P6, P9 or P21. Both strain were from a C57BL/6 background.

Wild animals	n/a
Field-collected samples	n/a
Ethics oversight	All animal procedures were performed according to the Canadian Council on Animal Care ethical regulations and were approved by the McGill University Research and Ethics Animal committee.

Note that full information on the approval of the study protocol must also be provided in the manuscript.
